# Transcriptional Analysis of Immunohistochemically Defined Subgroups of Non-Muscle-Invasive Papillary High-Grade Upper Tract Urothelial Carcinoma

**DOI:** 10.3390/ijms20030570

**Published:** 2019-01-29

**Authors:** Minsun Jung, Jeong Hoon Lee, Bohyun Kim, Jeong Hwan Park, Kyung Chul Moon

**Affiliations:** 1 Department of Pathology, Seoul National University College of Medicine, Seoul 03080, Korea; jjunglammy@gmail.com (M.J.); cherish1107@naver.com (B.K.); hopemd@hanmail.net (J.H.P.); 2 Seoul National University Biomedical Informatics (SNUBI), Division of Biomedical Informatics and Systems Biomedical Informatics National Core Research Center, Seoul National University College of Medicine, Seoul 03080, Korea; sosal@snu.ac.kr; 3 Department of Pathology, SMG-SNU Boramae Medical Center, Seoul 03080, Korea; 4 Kidney Research Institute, Medical Research Center, Seoul National University College of Medicine, Seoul 03080, Korea

**Keywords:** upper tract urothelial carcinoma, carcinoma, papillary, papillary urothelial carcinoma, carcinoma, transitional cell, RNA, messenger, transcriptome, gene expression, gene expression profiling, immunohistochemistry

## Abstract

Immunohistochemical (IHC) staining for CK5/6 and CK20 was reported to be correlated with the prognosis of early urothelial carcinoma in a way contrary to that of advanced tumors for unknown reasons. We aimed to characterize the gene expression profiles of subgroups of non-muscle-invasive papillary high-grade upper tract urothelial carcinoma (UTUC) classified by CK5/6 and CK20 expression levels: group 1 (CK5/6-high/CK20-low), group 2 (CK5/6-high/CK20-high), and group 3 (CK5/6-low/CK20-high). Expression of group 3 was predictive of worse prognosis of non-muscle-invasive papillary high-grade UTUC. Transcriptional analysis revealed 308 differentially expressed genes across the subgroups. Functional analyses of the genes identified cell adhesion as a common process differentially enriched in group 3 compared to the other groups, which could explain its high-risk phenotype. Late cell cycle/proliferation signatures were also enriched in group 3 and in some of the other groups, which may be used as a prognostic biomarker complementary to CK5/6 and CK20. Group 2, characterized by low levels of genes associated with mitogen-activated protein kinase and tumor necrosis factor signaling pathways, was hypothesized to represent the least cancerous subtype considering its normal urothelium-like IHC pattern. This study would facilitate the application of easily accessible prognostic biomarkers in practice.

## 1. Introduction

Recent advances in genetic and bioinformatic technologies have increased our understanding of the molecular characteristics of urothelial carcinoma. Based on gene expression profiles, it was demonstrated that both muscle-invasive (MIBC) and non-muscle-invasive urinary bladder carcinoma (NMIBC) can be divided into intrinsic molecular subtypes [1,2,3,4,5,6]. Although the classifications of MIBC were named differently by respective research groups, they had significant overlap: common luminal and basal molecular subtypes were reported [7]. Luminal type tumors expressed terminal differentiation markers of the urothelium, such as *KRT20*, *PPARG*, *GATA3*, *FOXA1*, and *UPK*s [2,5]. The basal type was enriched with basal cytokeratin, squamous differentiation, and stem cell signature markers, including *KRT5*, *KRT6*, *KRT14*, and *CD44* [2,3,5]. Molecular subtypes showed differences in prognosis, responsiveness to neoadjuvant chemotherapy, and targetable mutations; therefore, they would significantly influence MIBC treatment [5,8,9,10]. On the other hand, gene expression profiles of NMIBC showed 3 different clusters, named as class 1, class 2, and class 3, which differed in biologic signatures and in prognosis [6]. For example, class 1 tumors had high expression of early cell cycle genes and luminal type markers, class 2 was enriched with a late cell cycle signature and with the luminal type genes, and class 3 was characterized by high expression of basal type genes and long non-coding RNAs [6]. Despite elevated levels of luminal type markers, including *KRT20*, class 2 tumors showed poor prognosis, which indicated that class 2 may represent a molecular shift toward muscle-invasive carcinoma by taking the carcinoma in situ (CIS) pathway [6]. In another study on stage Ta urinary bladder carcinoma, cancers were segregated by copy number alteration profiles into two subtypes which varied in mammalian target of rapamycin complex 1 activity, metabolic pathways, and mutations [11].

Upper tract urothelial carcinoma (UTUC) is much less common than urinary bladder carcinoma, accounting for 5%–10% of total urothelial carcinoma, with its incidence estimated at 1/50,000 in the Western population [12]. UTUC showed high mortality rate with 5-year survival <50% for muscle invasive tumor and <10% for pT4 stage disease [12]. UTUC was revealed to have comparable types of mutations as urinary bladder carcinoma by targeted sequencing, but with different prevalence [13]. Comparison of public microarray data, including 12 UTUC and 20 urinary bladder carcinomas, demonstrated that both tumors were unidentifiable at the gene expression level, except for pT3 tumors [14]. It was also reported that UTUC was more enriched with luminal type genes compared with urinary bladder carcinoma [10,14]. However, the genomic landscape of UTUC has not been fully elucidated due to its rarity [15].

Several studies reported biomarkers of urothelial carcinoma using immunohistochemical (IHC) staining by reflecting its molecular frameworks, which were suggested to be relevant to prognosis and responsiveness to systematic treatment [16]. Luminal and basal type urinary bladder carcinoma were positive for luminal (CK20, GATA3, and uroplakin) and basal (CK5/6, CK14, and CD44) IHC markers, respectively [2,17]. In MIBC, positivity for CK5/6 and negativity for CK20 were correlated with poor survival [2]. In addition, high expression of CK5/6 and CK14 combined with low expression of FOXA1 and GATA3 in IHC staining was known to indicate a subtype with aggressive behavior or a basal/squamous-like (BASQ) subtype [18]. Interestingly, subgroups of non-muscle-invasive UTUC classified by CK5/6 and CK20 immunoprofiles showed contradicting prognoses to those of MIBC: CK5/6-low/CK20-high early UTUC had the worst outcome [19,20]. As a result, these IHC-defined subgroups of early UTUC supposedly have unique genetic features, the understanding of which would aid in comprehending the underlying pathobiology of non-muscle-invasive UTUC and facilitate the implementation of these IHC-based risk-stratifications in practice. Here, we analyzed the gene expression profiles of non-muscle-invasive papillary high-grade UTUC and revealed that CK5/6 and CK20 expression was not entirely relevant to molecular subtypes of this tumor. Instead, we postulated that cell adhesion and late cell cycle/proliferation processes were important to clinical behavior of subgroups defined by IHC staining for CK5/6 and CK20. In addition, tumors with CK5/6-high/CK20-high expression were suggested as a less cancerous subgroup.

## 2. Results

### 2.1. Subgroups of Non-Muscle-Invasive High-Grade Papillary UTUC Based on IHC Staining for CK5/6 and CK20

**Figure 1:**
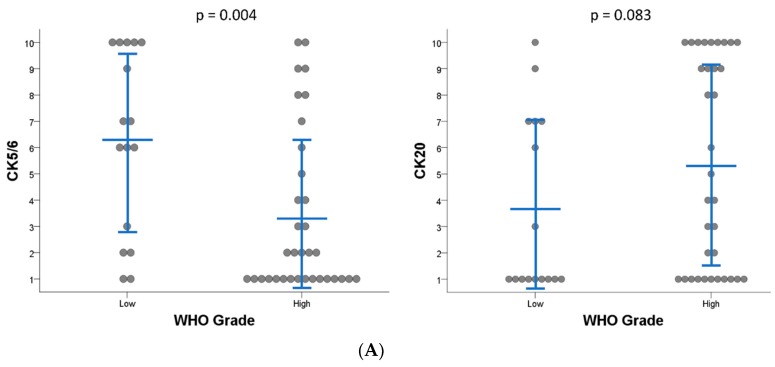

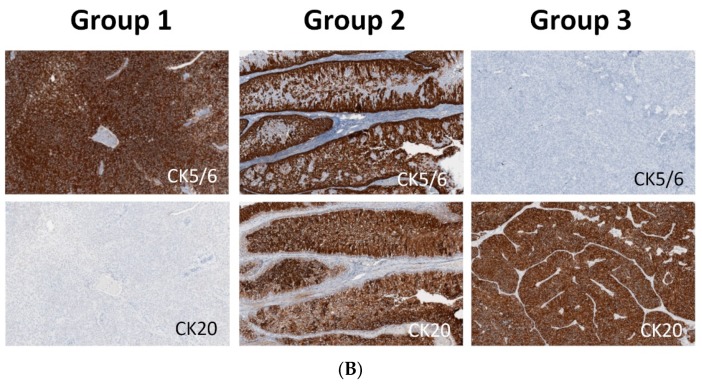
IHC staining for CK5/6 and CK20 in non-muscle-invasive papillary upper tract urothelial carcinoma (UTUC). (**A**) CK5/6-low and CK20-high IHC staining is significantly related to high WHO grade of non-muscle-invasive papillary UTUC. Blue bars indicate the mean value ± standard deviation. (**B**) Representative images of IHC staining for CK5/6 and CK20 across subgroups of non-muscle-invasive papillary high-grade UTUC. Original magnification × 40.

IHC staining for CK5/6, CK20, CK14, GATA3, FOXA1, CD44, and p53 was carried out for 50 non-muscle-invasive papillary UTUC tumors (Table S1). CK14 expression was almost absent in 98% of evaluable cases. By contrast, 95% and 76% of evaluable cases stained for GATA3 and FOXA1 in >80% of tumor cells, respectively. Correlations of IHC staining with pT stage, WHO grade, and the presence of CIS were analyzed. Among these characteristics, only WHO grade showed a significant association with IHC profiles: high WHO grade was associated with low CK5/6 (*p* = 0.004) and CD44 (*p* = 0.048), marginally with high CK20 (*p* = 0.083) and p53 (*p* = 0.051) expression, but not with CK14 (*p* = 1.000), GATA3 (*p* = 0.339), and FOXA1 (*p* = 0.778) staining (Figure 1A and Figure S1).

Further analysis was limited to high-grade tumors to remove grade-related genetic diversity [16]. We tried to classify non-muscle-invasive papillary high-grade UTUC using IHC staining for CK5/6 and CK20 as surrogate markers, which were known to be related to molecular subtypes and to the prognosis of UTUC and of urinary bladder carcinoma [19,20,21,22]. Fresh tissue with high (IHC score ≥ 6) or low (IHC score = 1) CK5/6 and CK20 expression was selected for RNA sequencing (RNA-seq). The same IHC criteria were marginally correlated with the progression-free survival (PFS) of the patients with non-muscle-invasive papillary high-grade UTUC (*p* = 0.071) retrieved from the published cohort [20]: tumors with CK5/6-low/CK20-high expression tend to show the worst PFS (Figure S2). Finally, three subgroups were established as follows: group 1, CK5/6-high/CK20-low; group 2, CK5/6-high/CK20-high; and group 3, CK5/6-low/CK20-high (Figure 1B). CK5/6 and CK20 were frequently stained in the whole layer of tumors in groups 1 and 3, respectively, without noticeable compartmentalization (Figure 1B). Although group 2 tumors were positive to both CK5/6 and CK20 in >50% of tumor cells, the expression of CK5/6 and CK20 was accentuated in basal and luminal cells, leaving out at least one cell layer of the basal and luminal portion, respectively, in all cases (Figure 1B). Clinicopathological data of the patients are summarized in Table 1. The median age of the patients was 69 years (range, 56–84) and the male/female sex ratio was 2:1. The tumors measured 3.6 ± 3.24 cm (mean ± standard deviation) in maximal diameter. Six (40%) patients were in stage pTa, and the other 9 (60%) patients were in pT1. CIS was observed in 5 cases (33.3%). There were no significant differences in clinicopathological parameters or in IHC profiles among the subgroups, except for CK5/6 and CK20 expression (Table 1). One sample that belonged to group 1 showed 20%–30% positivity for CK14. With the exception of this case, IHC staining for GATA3 and FOXA1 showed diffuse staining in all samples.

### 2.2. Identification of Differentially Expressed Genes (DEGs) between the Subgroups

**Figure 2:**
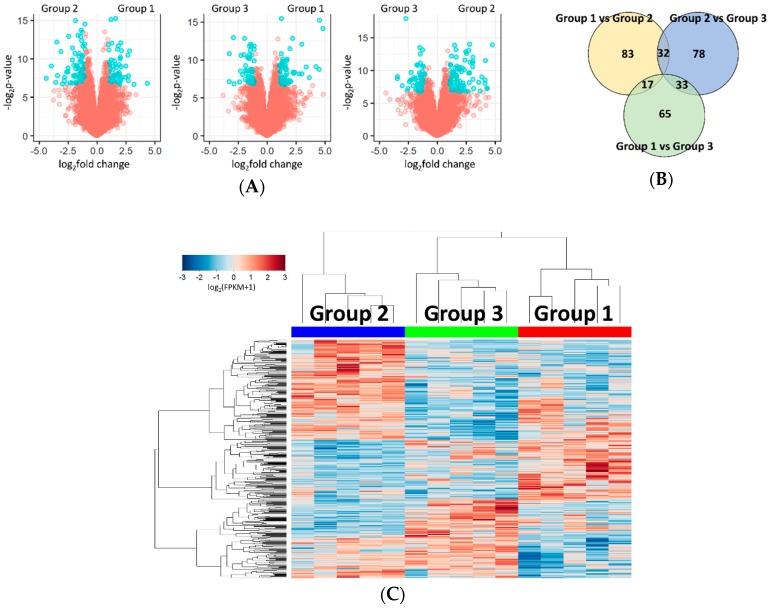
Identification of differentially expressed genes (DEGs) between the subgroups of non-muscle-invasive papillary high-grade UTUC. (**A**) Volcano plots of the DEGs between each subgroup. Genes that meet the threshold are in blue. (**B**) Venn diagram of all comparisons demonstrates 308 DEGs across the subgroups. (**C**) Unsupervised hierarchical clustering of all DEGs for all samples.

Using *p*-value <0.01 and |fold change| >2 as the cut-offs, 132, 115, and 143 DEGs were identified between groups 1 and 2, groups 1 and 3, and groups 2 and 3, respectively (Figure 2A,B and Table S2). Group 1 had 58 upregulated and 74 downregulated genes compared to group 2. Group 3, relative to group 1 and 2, harbored increased levels of 57 and 58 genes and decreased levels of 52 and 91 genes, respectively. Consequently, a total of 308 DEGs across all comparisons were found. They were put into unsupervised hierarchical clustering that demonstrated the congregation of the subgroups defined by IHC staining (Figure 2C). Principal component analysis of the DEGs revealed that tumors with similar CK5/6 and CK20 expression profiles made clusters separated from one another in 2D space (Figure S3).

### 2.3. Functional Analysis of the Subgroups

**Figure 3:**
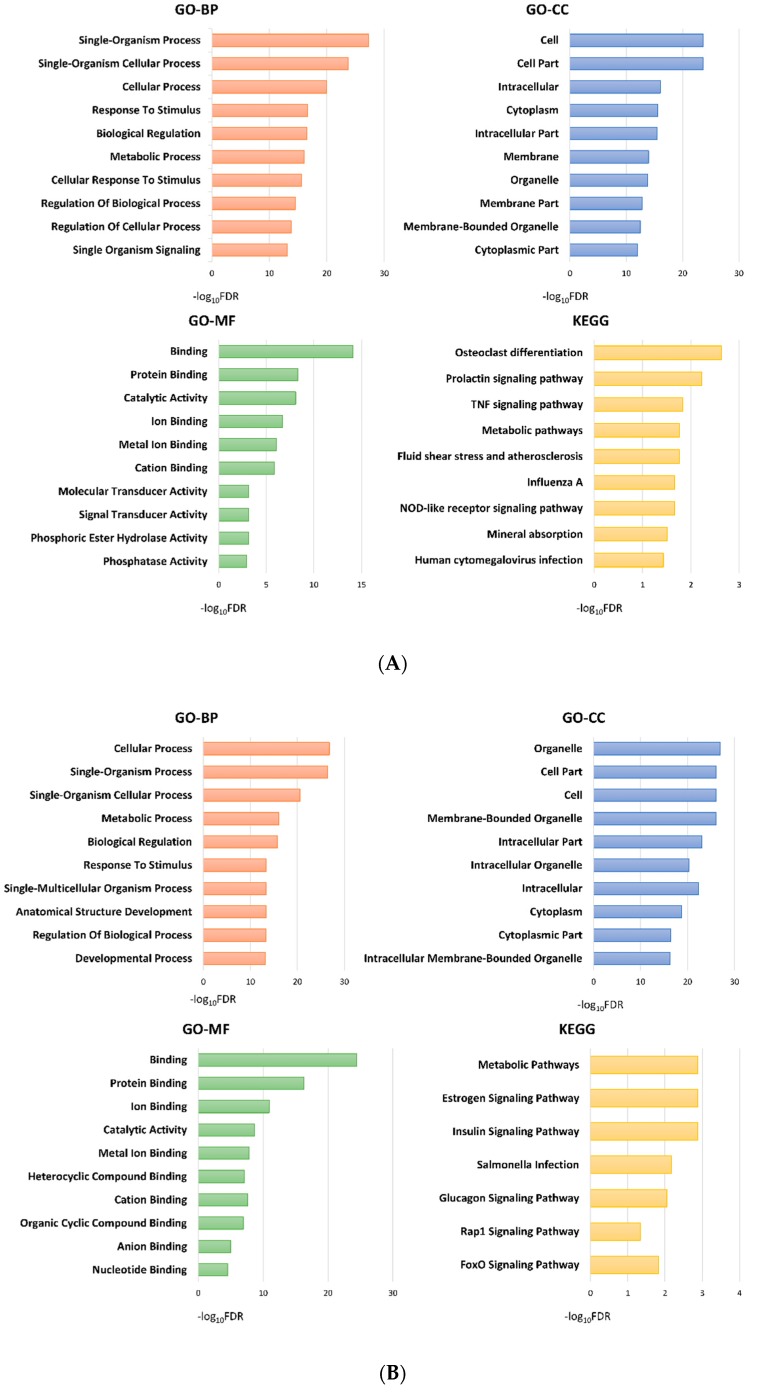

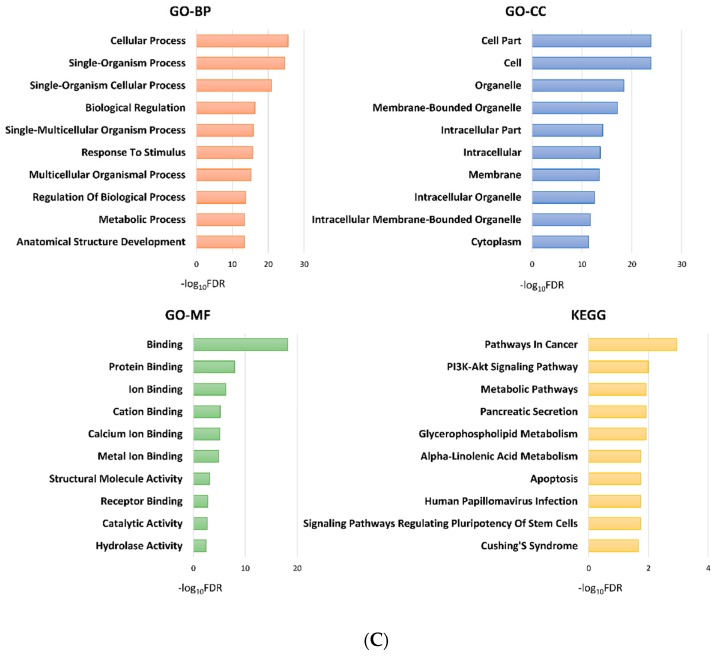
Top 10 significantly enriched biologic process (BP), cellular component (CC), and molecular function (MF) terms of gene ontology (GO) and Kyoto Encyclopedia of Genes and Genomes (KEGG) analyses of each comparison. (**A**) Group 1 vs. Group 2, (**B**) Group 1 vs. Group 3, (**C**) Group 2 vs. Group 3. FDR, false discovery rate.

Figure 3 shows the top 10 significant (false discovery date (FDR) < 0.05) results of gene ontology (GO) and Kyoto Encyclopedia of Genes and Genomes (KEGG) pathway analyses of DEGs between the subgroups.

#### 2.3.1. Group 3 was Enriched for Processes Related to Cellular Adhesion and Motility

**Figure 4:**
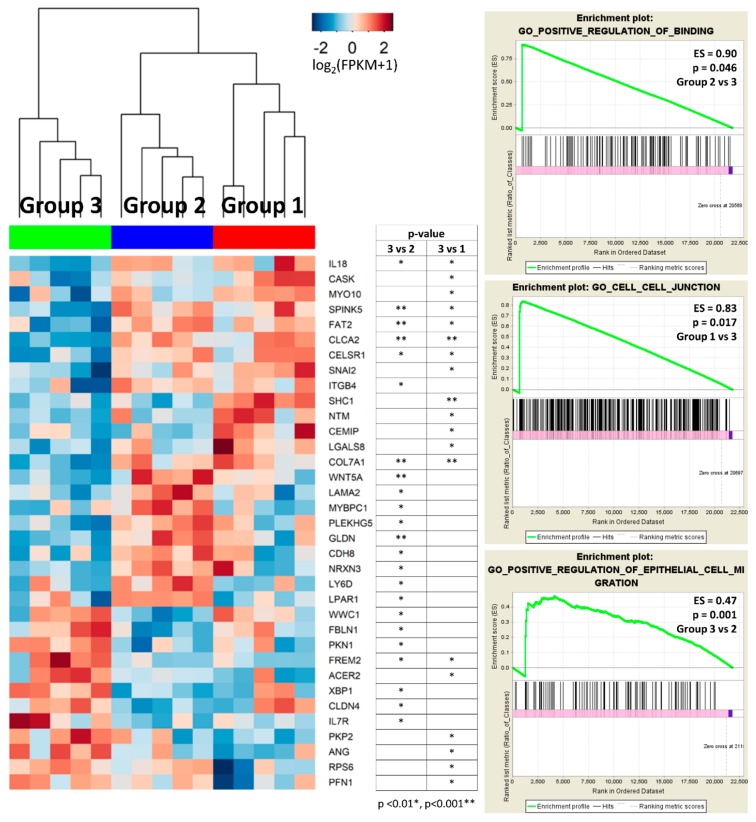
Heatmaps of DEGs enriched in cellular adhesion/motility processes and related GSEA results. Group 3 was enriched with biologic themes of cellular adhesion and motility. ES, enrichment score.

**Figure 5:**
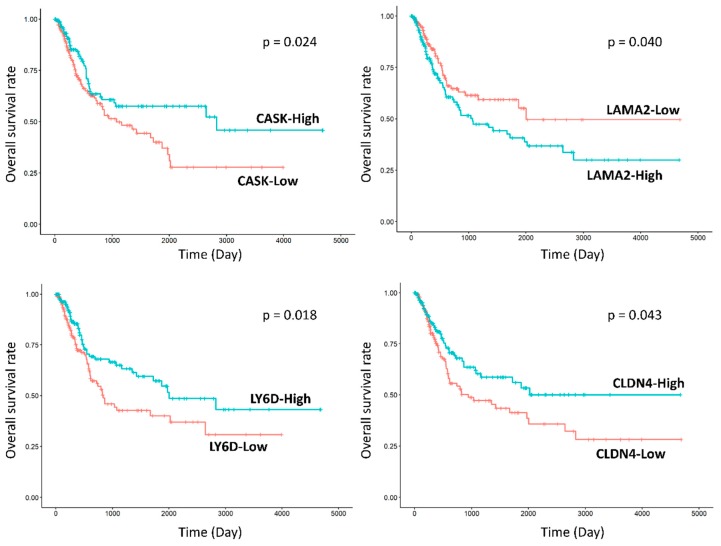
Survival analyses of DEGs involved in cellular adhesion/motility in The Cancer Genome Atlas (TCGA) database. Four genes were significantly associated with the overall survival of the patients with urinary bladder carcinoma.

Notably, DEGs between groups 3 and 1, or between groups 3 and 2 were enriched in cell adhesion and motility in GO analyses, including ‘cell adhesion’ (FDR < 0.001), ‘biological adhesion’ (FDR < 0.001), ‘cell–cell adhesion’ (FDR < 0.001), ‘regulation of cell adhesion’ (FDR = 0.015), and ‘cell junction’ (FDR = 0.021) between groups 3 and 1, and ‘cell adhesion’ (FDR < 0.001), ‘biological adhesion’ (FDR < 0.001), ‘cell–cell adhesion’ (FDR < 0.001), ‘cell–cell adhesion via plasma-membrane adhesion molecules’ (FDR = 0.028), ‘cell motility’ (FDR = 0.045), and ‘regulation of cell adhesion’ (FDR = 0.046) between groups 3 and 2. These processes involved several DEGs, for example, *CASK*, *MYO10*, *SPINK5*, *FAT2*, *CLCA2, CELSR1, SHC1*, *LGALS8*, *COL7A1*, *WNT5A*, *LAMA2*, *CDH8*, *NRXN3*, and *LY6D* that were downregulated, and *WWC1*, *PKN1*, *FREM2*, *XBP1*, *CLDN4*, *PKP2*, *ANG*, and *PFN1* which were upregulated in group 3 compared to the other subgroups (Figure 4). Gene set enrichment analysis (GSEA) confirmed the alteration of cellular binding/junction/migration signatures in group 3, including diminished function of binding and enhanced function of cell migration (Figure 4). Furthermore, GO analysis of DEGs between groups 3 and 1 showed enrichment of junctional complexes, such as ‘adherens junction’ (FDR = 0.005)’ and ‘anchoring junction’ (FDR = 0.006). Finally, we tried to validate correlation of these DEGs related to cellular adhesion and motility with the prognoses of patients with urothelial carcinoma in The Cancer Genome Atlas (TCGA) database. As a result, expression levels of *CASK* (*p* = 0.024), *LAMA2* (*p* = 0.040), *LY6D* (*p* = 0.018), and *CLDN4* (*p* = 0.043) were significantly associated with the overall survival of patients with urinary bladder carcinoma (Figure 5). Finally, KEGG analysis of DEGs between groups 3 and 2 showed enrichment of ‘pathways in cancer’ (FDR = 0.001) and ‘PI3K-Akt signaling pathway’ (FDR = 0.010) (Figure 3C).

#### 2.3.2. Group 2 Showed Downregulation of Mitogen-Activated Protein Kinase (MAPK) and Tumor Necrosis Factor (TNF) Signaling Pathways

**Figure 6:**
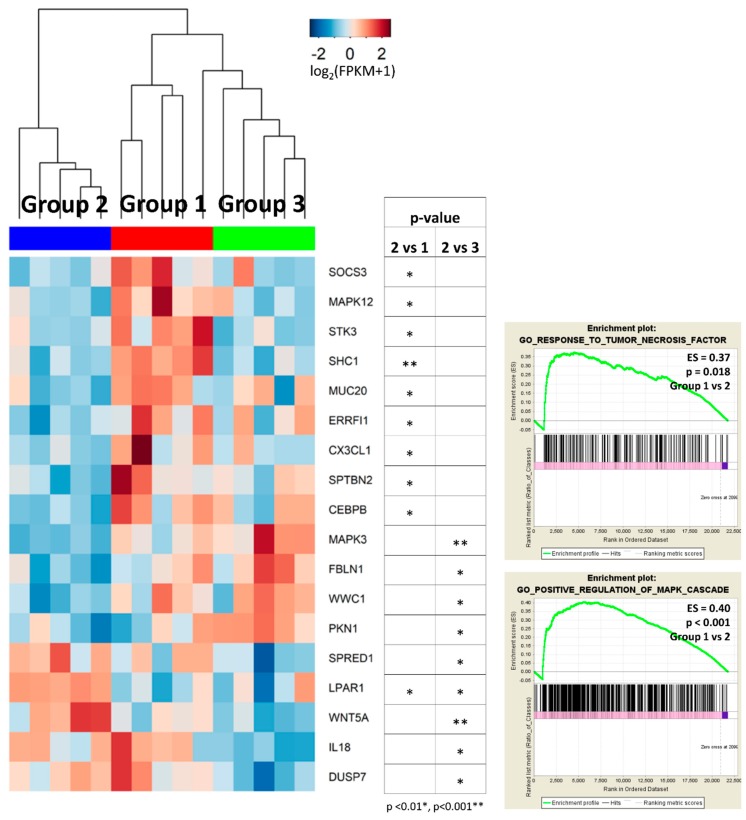
Heatmaps of DEGs enriched in MAPK or TNF signaling pathways, along with related GSEA results. Group 2 was characterized by low levels of genes involved in MAPK or TNF signaling pathways, except for a few genes that play regulatory roles in these pathways. ES, enrichment score.

Group 2 showed enrichment of MAPK process in GO analysis, compared to group 1 (‘MAPK cascade’, FDR = 0.044) and to group 3 (‘activation of MAPK activity’, FDR = 0.009; ‘regulation of MAP kinase activity’, FDR = 0.012; ‘MAPK cascade’, FDR = 0.018; ‘regulation of MAPK cascade’, FDR = 0.040). In addition, KEGG pathway analysis revealed that DEGs between groups 1 and 2 were enriched in TNF signaling pathway (FDR = 0.014) (Figure 3A). Most DEGs associated in these pathways were downregulated in group 2 tumors (Figure 6). GSEA also indicated group 2 tumors were less reactive to MAPK and TNF signaling cascades than group 1 tumors (Figure 6).

#### 2.3.3. Expression of Biologic Signature Genes of the Subgroups 

**Figure 7:**
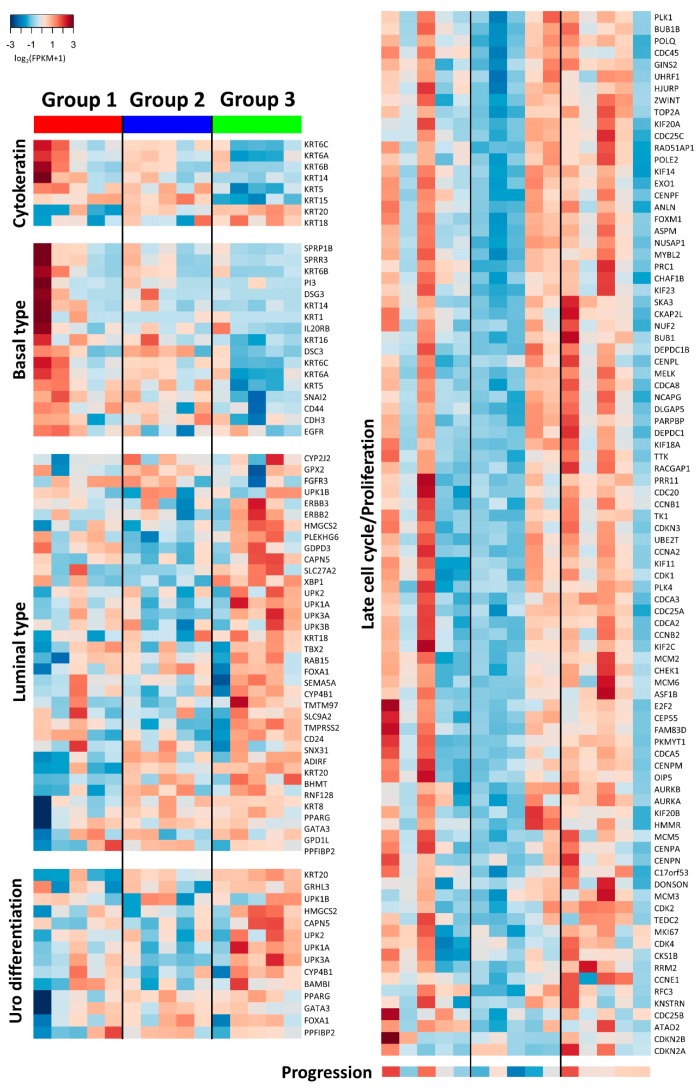
Heatmaps of known biological signature genes extracted from previous reports, with samples arranged in each column and subgroups divided by black vertical lines. Progression (right bottom) was visualized according to the integrated score (red, high; blue, low). Uro differentiation, urothelial differentiation.

To further delineate the properties of the IHC-based subgroups, we evaluated the expression of known biologic markers (Figure 7) [1,3,6,23,24,25]. Each subgroup was enriched with different types of keratin: *KRT*5, *KRT*6, *KRT*14, and *KRT*15 in group 1, *KRT18* and *KRT20* in group 3, and all in group 2. The expression patterns of basal and luminal type markers clustered moderately with group 1 and group 3, respectively. Group 2 showed modest enrichment of both subtype markers. Urothelial differentiation markers, sharing some of the gene sets with those of luminal type, showed moderate overexpression in group 3. Gene expression of late cell cycle/proliferation signature overlapped substantially with group 3 and moderately with some of the other groups, which was also differentially enriched between groups 3 and 1 (‘cell proliferation’, FDR = 0.042) and between groups 2 and 1 (‘regulation of cell proliferation’, FDR = 0.012; ‘cell proliferation’, FDR = 0.025) in GO analyses. Consistently, the expression of genes related to the progression of early urothelial carcinoma [26] was higher in group 3 and 1 than in group 2 (Mann–Whitney *U*, *p* = 0.028), showing a huge parallel to that of late cell cycle/proliferation genes, in that it was high in group 3 and in two samples of group 1. The two group 1 tumors with elevated levels of cell cycle/proliferation genes tend to express *KRT14* more than the others, and the tumor with the highest *KRT14* level was the only one positive for CK14 by IHC staining. Expression levels of early cell cycle, epithelial-to-mesenchymal transition, and cancer stem cell markers did not significantly overlap with the subgroups based on IHC staining. Finally, independent molecular classifiers of MIBC and NMIBC were applied to the present tumors [3,6]. Although they yielded gene expression clusters similar to their original designs, they did not overlap with the present IHC-defined subgroups (Figure S4).

## 3. Discussion

In the present study, we performed transcriptional investigation on non-muscle-invasive papillary high-grade UTUC by comparing its subgroups defined by CK5/6 and CK20 expression. First, we showed that immunoreactions of low CK5/6, high CK20, low CD44, and high p53 were predictive of high WHO grade in non-muscle-invasive papillary UTUC [19,20,27]. The 4 IHC markers defining the BASQ subtype of MIBC, CK14-positive, CK5/6-positive, GATA3-negative, FOXA1-negative expression [18] were not readily applicable to non-muscle-invasive papillary UTUC because positivity for CK14 and negativity for GATA3 or for FOXA1 was rarely observed in this tumor. To minimize molecular divergence between low- and high-grade urothelial carcinoma [16], we excluded low-grade tumors and focused on high-grade tumors. Along with previous studies [19,21,28], CK5/6-low/CK20-high expression was marginally correlated with the poor PFS of non-muscle-invasive papillary high-grade UTUC, which was demonstrated in the patients from the published cohort [20]. Therefore, IHC staining of CK5/6 and CK20 was adapted as prognostic markers of non-muscle-invasive papillary high-grade UTUC, which could be relevant to its molecular classifications [2,5,19,20,22]. To ensure the protein expression of the RNA-seq samples, expression levels of CK5/6 and CK20 were determined based on IHC staining on adjacent tissue with strict cut-offs [29]. Consequently, mRNA levels of basal/intermediate and luminal keratins concurred with the IHC staining profiles of CK5/6 and CK20, overall [30].

CK5/6 and CK20 expression has been suggested to represent the differentiation status of urothelium; therefore, IHC staining for these proteins can be useful as a surrogate marker pertinent to molecular subtypes of urothelial carcinoma [2,5]. CK5/6 is expressed in basal and progenitor cells of the normal urothelium which is conserved in basal type urothelial carcinoma that supposedly obtains a basal cell-like undifferentiated phenotype [31,32]. On the other hand, high levels of CK20 expression in urothelial carcinoma, as normally observed in terminally differentiated urothelium, are suggested to indicate neoplasm in well-differentiated states [31]. These were well-correlated with prognostic differences of basal and luminal type MIBC [1,2,3,5,24,25,31,33,34]. Consistent with the correlation of keratin expression and basal/luminal types of MIBC, early urothelial carcinomas showing CK5/6-high/CK20-low and CK5/6-low/CK20-high immunoreactions were enriched for basal type and luminal type/urothelial differentiation signature genes, respectively, in this study and in the previous one [1]. However, the contrasting clinical outcomes associated with CK5/6 and CK20 expression, assessed at the protein or at the mRNA levels, were frequently reported in early urothelial carcinoma of the upper or lower tract [6,19,20,21] and, occasionally, in MIBC [22]. Furthermore, previous classifiers of urothelial carcinoma based on gene expression were assigned to the present cases in a way that was independent of their IHC staining [3,6]. Collectively, these results implied that distinct molecular frameworks, that would determine their respective behaviors, were inherent in the present subgroups rather than the basal vs. luminal paradigm.

Cell adhesion and motility, in agreement with other reports [1,33], was the distinct function that was differentially enriched between groups 3 and 1 and between groups 3 and 2. Of note, group 3 showed downregulation of several genes involved in major cell adhesion complexes, including adherens junction (*CDH8*, *CLCA2*) [35,36,37], desmosome (*SPINK5*) [38], focal adhesion (*MYO10*, *SHC1*) [39,40], and basement membrane and the interaction with it (*LGALS8*, *LAMA2*, *COL7A1, ITGB4*) [41,42], with tight junction (*CLDN4*) being the exception [43], which would promote migration and invasion of cancer cells. As suggested by Sjodahl et al. [1], the major high-risk subtype of NMIBC, ‘genomically unstable’, had low expression levels of genes engaged in major adhesion structures, such as adherens junction, desmosome, and gap junction, with the exception being tight junction (*CLDN*s), compared to the low-risk subtype, ‘urothelial-like A’. The ‘genomically unstable’ subtype showed IHC staining for CK5 and CK20, similar to that of group 3 in this study [1,33]. In line with this, functional enrichment analyses and GSEA supported the premise that the loss of non-apical cell-to-cell and cell-to-matrix adhesions is a major determinant of the high-risk subtype, group 3 [37]. In addition, the non-canonical Wnt/PCP pathway (*FAT2*, *CELSR1*, *WNT5A*), known to suppress early-staged cancers by regulating cell adhesion or migration, was downregulated in group 3 [44]. This was also substantiated by the results showing that the alteration of the genes observed in group 3 have been reported to promote invasion of and to be associated with the poor prognoses of urothelial carcinoma and other various malignancies, including downregulation of *CLCA2* [36], *SPINK5* [45], *SHC1* [40], *LGALS8* [42], *COL7A1* [41], *FAT2* [44], *WNT5A* [44], and *NRXN3* [46], and upregulation of *CLDN4* [43], *PKN1* [47], *FREM2* [48], *XBP1* [49,50], *PKP2* [51], *ANG* [52], and *PFN1* [53]. We tried to validate the prognostic values of these genes in the independent TCGA urinary bladder carcinoma cohort. However, their prognostic consequences were inconsistent, and a few of those genes altered in group 3 even predicted longer survival in the TCGA database. This result, in conjunction with those of previous studies [35,41,44,53], suggested that the adhesion molecules may act differently in advanced disease, which accounted for 99.2% of the TCGA cohort. In treatment of non-muscle-invasive UTUC, adhesion molecules differentially expressed among the present subgroups provide candidates for targeted therapy [44,54], which warrants a deeper investigation.

Late cell cycle/proliferation signature genes were predominantly upregulated in most group 3 tumors, as well as in a few samples of the other subgroups, consistent with the molecular subtypes of NMIBC with poor prognosis suggested in previous reports, ‘genomically unstable’, ‘urothelial-like B’, and ‘squamous cell carcinoma-like’ subtypes, which shared elevated levels of these signature genes compared to the ‘urothelial-like A’ subtype [1,6,24,33]. High levels of late cell cycle/proliferation markers were also observed in aggressive subtypes of MIBC [6,24]. In addition, IHC staining for cyclin B1, as a late cell cycle marker, combined with CK5 expression and tumor histology in risk-stratified patients with T1 NMIBC [33,34]. Moreover, we showed that the enrichment pattern of late cell cycle/proliferation correlated closely with that of the NMIBC progression signature [26]. Therefore, it is feasible to postulate that the enhanced cell proliferation of group 3 would support its aggressive phenotype, which was possibly obtained by bypassing cell cycle checkpoints [1]. In addition, different molecular subtypes of NMIBC that showed diverse clinical behaviors, ‘urothelial-like A’, ‘urothelial-like B’, and ‘squamous cell carcinoma-like’, as suggested by Sjodahl et al., included tumors with CK5/6-high/CK20-low IHC staining [1,33,34], which was representative of group 1. Therefore, group 1 is suggested to contain prognostically heterogeneous tumors. Considering the remarkable overexpression of these signature genes observed in certain tumors of group 1, genes related to late cell cycle/proliferation are expected to be used as risk-stratification markers within group 1 tumors. Likewise, they would also be useful for group 2 tumors that showed modest discrepancies in the level of late cell cycle/progression signature within the subgroup. This seemed analogous to the ‘urothelial-like A’ and ‘urothelial-like B’ subtypes, as both subtypes could be positive for CK5/6 and CK20, similar to group 2 tumors [1,33]. Alternatively, IHC staining for CK14, observed only in one of the group 1 samples, was one of the characteristics of the ‘squamous cell carcinoma-like’ subtype [33]; therefore, CK14 immunostaining could be used for subclassifying group 1 tumors, even though it was rarely positive in non-muscle-invasive papillary high-grade UTUC. 

The accentuated patterns of IHC staining for CK5/6 and CK20 observed in group 2 were reminiscent of the immunoprofiles that were associated with low-risk phenotypes of superficial papillary urothelial neoplasms which maintained stratified CK5/6 and CK20 expression of the normal urothelium [20,55]. Similar IHC staining for CK5/6 and CK20 was also reported in the low-risk ‘urothelial-like A’ subtype which was well-differentiated at the gene expression level [1,33,34]. In transcriptional investigation, group 2 had moderate enrichment for genes involved in urothelial differentiation as well as in basal and luminal type signatures. Moreover, most DEGs enriched in MAPK or TNF pathways were expressed at lower levels in group 2 than the other groups, except for *SPRED1, DUSP7*, and *WNT5a*, that are known to downregulate MAPK and TNF signaling cascades, respectively [56,57]. MAPK activation was shown to be an important mediator of cellular transformation induced by constitutively activated *FGFR3*, which have crucial roles in tumorigenesis of papillary urothelial carcinoma [58]. In addition, TNF, a major inflammatory cytokine, is known to participate in many steps of carcinogenesis and tumor progression [59]. As a result, we hypothesize that group 2 represents the least cancerous subtype which maintains molecular structures of the normal urothelium, as exemplified by expression of *KRT*s; the expression of CK5/6 and CK20 in this group is not entirely representative of the basal and luminal types, respectively [60]. It is still not clear if this subgroup is a precursor of progressed lesions [6], for example, group 1 or group 3, or a distinct subtype with different pathogenesis.

This study had some limitations. The number of the patients was constrained by the strict cut-off criteria of IHC staining. Due to the short follow-up duration of the patients subjected to RNA-seq, their exact outcome was unevaluable, even though it was validated in the independent patient cohort. Finally, cellular adhesion, late cell cycle/proliferation signature, MAPK, or TNF signaling pathway genes, suggested as potential biological keys in the present and in previous studies, need external validation for confirmation. However, this study had the merits of investigating the transcriptional characteristics of subgroups defined by routinely used IHC staining, CK5/6 and CK20, which would facilitate the application of the accumulating genetic/phenotypic information of urothelial carcinoma, in practice.

In conclusion, IHC staining for CK5/6 and CK20 classifies subgroups of non-muscle-invasive papillary high-grade UTUC which are prognostically relevant but molecularly different from previous gene expression subtypes established in MIBC or even in NMIBC. Group 2 tumors were postulated to represent a less cancerous subtype.

## 4. Materials and Methods

### 4.1. Sample Selection and Immunohistochemistry

Radical nephroureterectomy specimens of Korean patients with UTUC were collected from Seoul National University Hospital from January 2016 to June 2018. Harvested tumors were divided in two. One half was snap-frozen and stored for RNA-seq, and the other was made into a formalin-fixed paraffin embedded (FFPE) block. Protein expression of frozen tissue was evaluated based on the IHC staining of its counterpart FFPE block. IHC staining was done on 4 μm-thick slides with the BenchMark Autostainer (Ventana, Tucson, AZ) according to the manufacturer’s instructions and using the following antibodies: CK5/6 (1:100; D5/16 B4; Dako, Glostrup, Denmark), CK20 (1:50; Ks 20.8; Dako), CK14 (1:300; LL002; Cell Marque, Rocklin, CA), GATA3 (L50-823; 1:500; Cell Marque), FOXA1 (1:500; PA5-27157; Thermo Fisher, Waltham, MA), CD44 (1:100; 156-3C11; Thermo Fisher), and p53 (1:1000; DO7; Dako). Immunoreactivity was scored semiquantitatively from 1 (<10%) to 10 (≥90%) by a 10% scale. Grading was based on the 2004 WHO system [12]. Lymph node involvement or distant metastasis was not found at diagnosis in any patients. The patients did not receive any neoadjuvant treatments before surgery. Cases showing divergent differentiation were excluded because of their potential aberrant reaction to CK20 [22]. Comparison of IHC profiles was performed by Mann–Whitney *U* test or Kruskal–Wallis H test. Correlation of clinicopathological variables with IHC-defined subgroups was calculated by Fisher’s exact test. SPSS 25 software (IBM, Armonk, NY, USA) was used for statistical analysis, with a two-tailed *p*-value < 0.05 considered significant. This study was approved by the Institutional Review Board of Seoul National University Hospital (H-1810-148-983, 07 November 2018).

### 4.2. Validation of Prognosis Associated with Immunohistochemical Profiles of RNA-Seq Subgroups

Prognostic validation of the IHC criteria of RNA-seq subgroups was performed by Kaplan–Meier and log-rank tests of the PFS of the patients with non-muscle-invasive papillary high-grade UTUC who were retrieved from the previously published cohort [20]. Protein expression of CK5/6 and CK20 was re-evaluated semiquantitatively in accordance with the IHC criteria of IHC-defined subgroups, high (≥ 50%) or low (<10%), from the triplicate tissue microarray IHC slides made for the previous study [20]. From 93 patients with non-muscle-invasive papillary high-grade UTUC, 39 tumors met this IHC staining criteria, including 13 CK5/6-high/CK20-low, 4 CK5/6-high/Ck20-high, and 22 CK5/6-low/CK20-high tumors. The median follow-up period was 60 months (range, 1–207). Disease progression was defined by local recurrence except for urinary bladder or distant metastasis. 

### 4.3. Transcription Analysis and Identification of Differentially Expressed Genes

mRNA was extracted using a punch with a plunger (3 mm in diameter) from the pure papillary tumor area (tumor cellularity >90%) of frozen tissue that was marked on a hematoxylin and eosin-stained frozen slide. RNA-seq was performed as previously described [61] using the Illumina HiSeq 2500 platform (Illumina, San Diego, CA, USA). Paired demultiplexed fastq files were generated, and initial quality control was performed using FastQC (Phred quality score >30) [62]. Adapter trimming was conducted using the Trimmomatic tool [63] followed by mapping to the human genome (UCSC hg19) using the HISAT2 and Bowtie2 [64]. Known transcripts were assembled with the StringTie tool [65]. Consequently, 21,780 genes were found across the samples. Quantile normalization was done on log_2_ (FPKM+1). To fit a linear model for each gene based on the three-group study design, the function of lmFit was used (limma) [66]. Then, the eBayes function was used to calculate the empirical Bayes moderated *t*-statistic.

### 4.4. Functional Enrichment Analysis

Functional enrichment of DEGs were found from GO [67] and KEGG pathway [68] database with FDR < 0.05 as a cut-off. The GSEA of all genes was used to interpret their functions from the public database [69]. Biological signatures were acquired from the respective publications [1,3,6,23,24,25]. The integrated score for the progression of NMIBC was calculated by subtracting the total value of *COL4A3BP*, *MBNL2*, *NEK1*, *FABP4*, and *SKAP2* from that of *KPNA2*, *BIRC5*, *UB2C*, *CDC25B*, *COL4A1*, *MSN*, and *COL18A1* [26].

### 4.5. TCGA Data Preprocessing and Survival Analysis

From the TCGA database, 399 samples of urinary bladder urothelial carcinoma were collected that contained mRNA expression and survival data. Most tumors were WHO high-grade (*n* = 375) and MIBC (*n* = 396). The median follow-up period was 1735 days, during which 109 patients died. Preprocessing of the mRNA expression data was performed in the R 3.4.3 statistical environment: mRNA expression level quantified by RSEM raw counts were normalized using the limma-voom, which applied linear modeling to voom-transformed read counts [70]. We determined the median value of gene expression as a cut-off point to divide patients into high- and low-expression groups. Kaplan–Meier and log-rank tests were applied for survival analysis.

## Figures and Tables

**Table 1:** Clinicopathological characteristics of subgroups of non-muscle-invasive papillary high-grade UTUC and their IHC expression.

Variables	Group 1	Group 2	Group 3	*p*-value
**Number (%)**	5 (100)	5 (100)	5 (100)	
**Age**				1.000
≥69	3 (60)	3 (60)	3 (60)	
<69	2 (40)	2 (40)	2 (40)	
**Sex**				1.000
Male	3 (60)	4 (80)	3 (60)	
Female	2 (40)	1 (20)	2 (40)	
**Size**				0.725
≥3.6	2 (40)	1 (20)	0 (0)	
<3.6	3 (60)	4 (80)	5 (100)	
**Organ**				0.301
Pelvis/calyx	4 (80)	1 (20)	3 (60)	
Ureter	1 (20)	4 (80)	2 (40)	
**Stage**				0.800
T1	4 (80)	2 (40)	3 (60)	
Ta	1 (20)	3 (60)	2 (40)	
**CIS**				1.000
Present	2 (40)	1 (20)	2 (40)	
Absent	3 (60)	4 (80)	3 (60)	
**IHC score (mean ± SD) ^1^**				
CK5/6	6.4 ± 2.41	7.4 ± 2.41	1 ± 0.00	0.001
CK20	1.0 ± 0.00	8.4 ± 1.52	10 ± 0.00	<0.001
CD44	6.0 ± 2.83	5.4 ± 3.21	3.0 ± 2.55	0.162
p53	7.5 ± 3.70	6.3 ± 2.22	4.8 ± 3.55	0.428
CK14	1.4 ± 0.89	1.0 ± 0.00	1.0 ± 0.00	1.000
GATA3	8.0 ± 3.94	10.0 ± 0.00	10.0 ± 0.00	0.286
FOXA1	6.4 ± 4.51	10.0 ± 0.00	8.4 ± 1.95	0.066

^1^ Semiquantitative scores from 1 (<10%) to 10 (90%–100%) by a 10% scale. CIS, carcinoma in situ; IHC, immunohistochemistry.

## Supplementary Materials

Supplementary materials can be found at http://www.mdpi.com/1422-0067/20/3/570/s1.

## Abbreviations

MIBC	muscle-invasive bladder cancer
NMIBC	non-muscle-invasive bladder cancer
CIS	carcinoma in situ
UTUC	upper tract urothelial carcinoma
IHC	immunohistochemistry
BASQ	basal/squamous-like
RNA-seq	RNA sequencing
PFS	progression-free survival
DEG	differentially expressed gene
FDR	false discovery rate
GO	gene ontology
KEGG	Kyoto Encyclopedia of Genes and Genomes
BP	biologic process
CC	cellular component
MF	molecular function
GSEA	gene set enrichment analysis
TCGA	The Cancer Genome Atlas
MAPK	mitogen-activated protein kinase
TNF	tumor necrosis factor
