# Supplementary material for: Transcriptional Analysis of Immunohistochemically Defined Subgroups of Non-Muscle-Invasive Papillary High-Grade Upper Tract Urothelial Carcinoma
Source: Int J Mol Sci. 2019 Jan 29;20(3):570. doi: 10.3390/ijms20030570 (PMC6386996; doi:10.3390/ijms20030570)
Supplement: Supplementary file 1 [file ijms-20-00570-s001.zip › Figure S1-S4_R1.pdf]

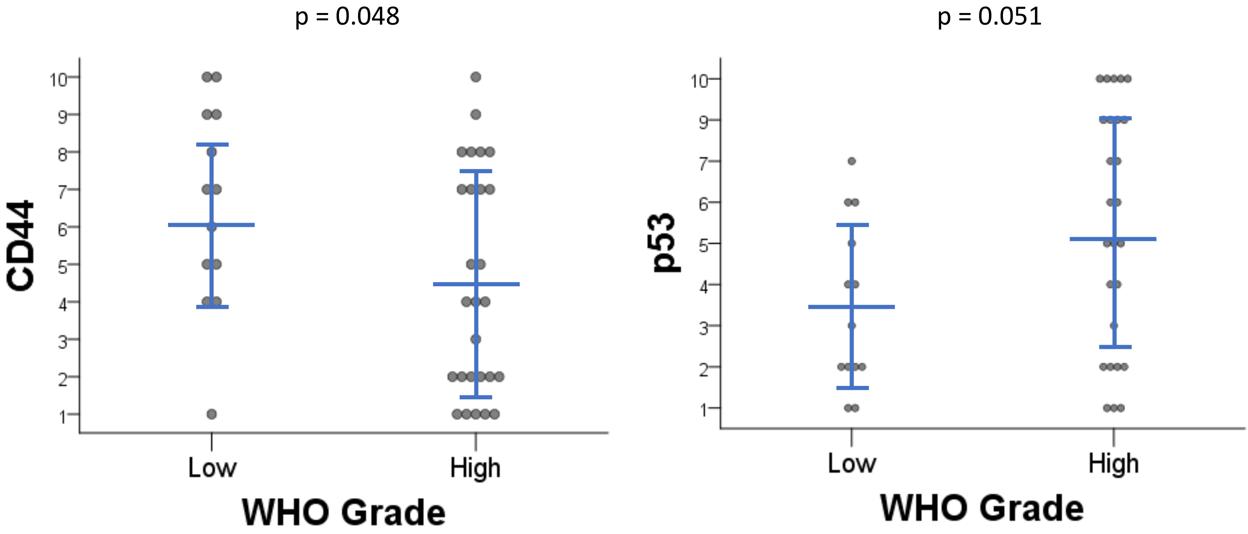

Figure S1. IHC staining for CD44 and p53 in non-muscle-invasive papillary UTUC. Blue bars indicate mean value with s.d..

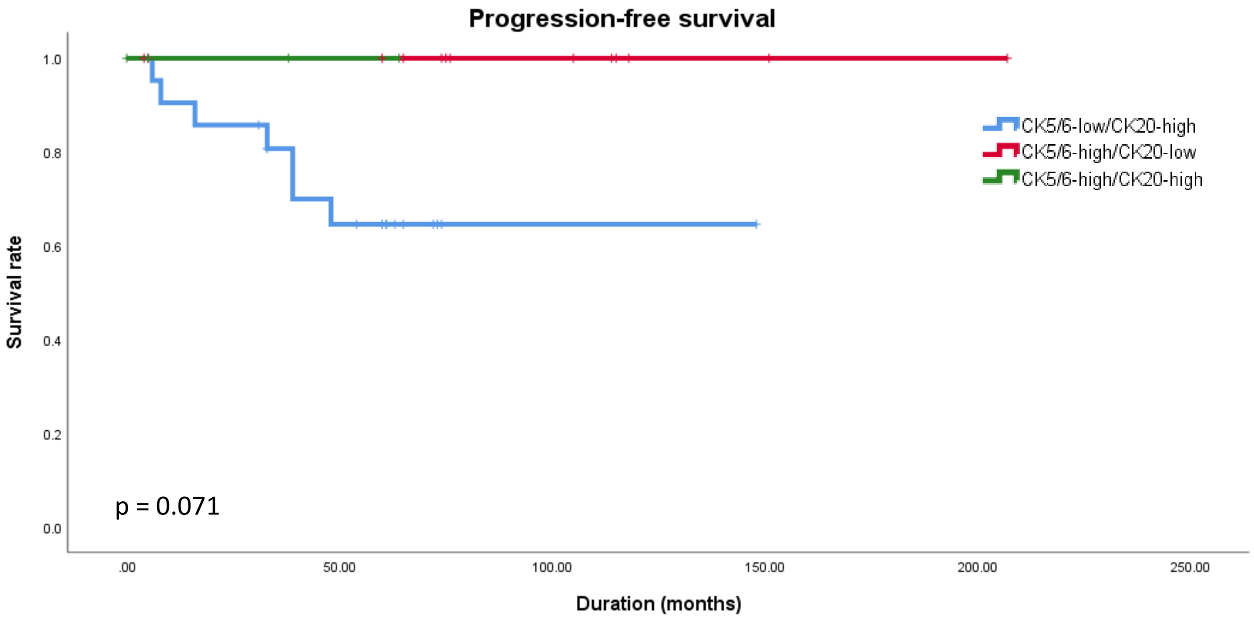

Figure S2. Kaplan-Meier and log-rank tests of IHC staining for CK5/6 and CK20 in non-muscle-invasive papillary high-grade UTUC. The IHC staining profile of Group 3, CK5/6-high/CK20-low expression, was marginally associated with shorter PFS period. IHC and survival data were retrieved from previous publication [20].

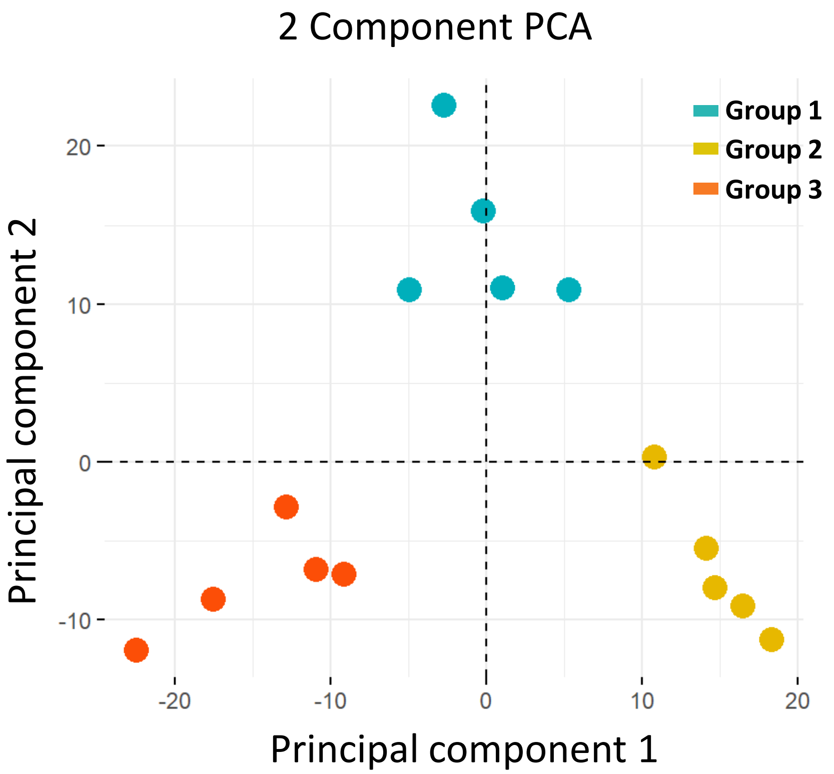

Figure S3. Principal component analysis of DEGs. Groups 1, 2, and 3 form distinct clusters.

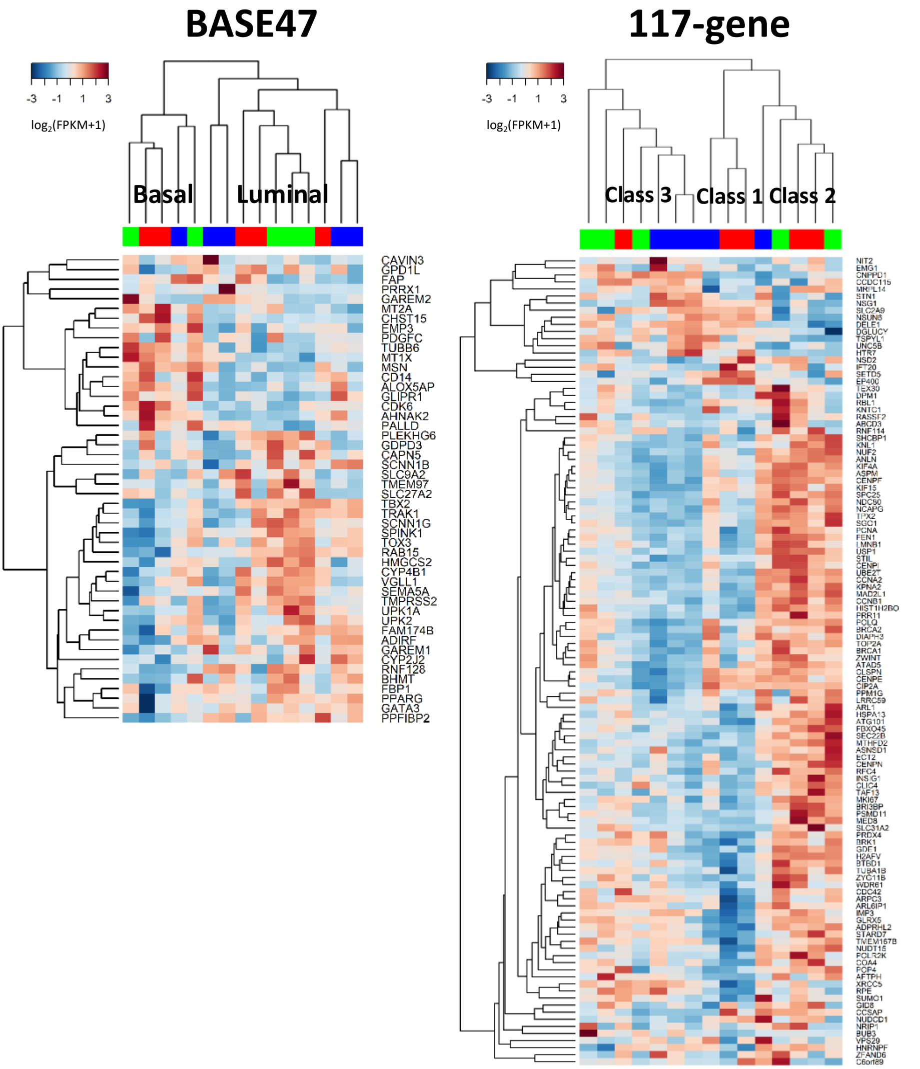

Figure S4. Unsupervised hierarchical clustering of BASE47 (left) [3] and 117-gene (right) [6] classifiers. All of BASE47 and 115 of 117 genes were present in the samples of this study. Color bars on the top indicate IHC-defined groups: red (group 1), blue (group 2), and green (group 3).
